# Prophylactic plasma and platelet transfusion in the critically Ill patient: just useless and expensive or even harmful?

**DOI:** 10.1186/s12871-015-0074-0

**Published:** 2015-06-09

**Authors:** Klaus Görlinger, Fuat H. Saner

**Affiliations:** Department of Anesthesiology and Intensive Care Medicine, University Hospital Essen, Essen, Germany; Tem International GmbH, Munich, Germany; Department of General, Visceral and Transplant Surgery, University Hospital Essen, Essen, Germany

**Keywords:** Critically ill, Tracheostomy, INR, Thromboelastometry, Prophylactic plasma transfusion, Patient blood management

## Abstract

It is still common practice to correct abnormal standard laboratory test results, such as increased INR or low platelet count, prior to invasive interventions, such as tracheostomy, central venous catheter insertion or liver biopsy, in critically ill patients. Data suggest that 30–90 % of plasma transfused for these indications is unnecessary and puts the patient at risk. Plasma transfusion is associated with a high risk of transfusion-associated adverse events such as transfusion-associated circulatory overload (TACO), transfusion-related lung injury (TRALI), transfusion-related immunomodulation (TRIM), and anaphylaxis/allergic reactions. Therefore, the avoidance of inappropriate plasma transfusion bears a high potential of improving patient outcomes. The prospective study by Durila et al., published recently in BMC Anesthesiology, provides evidence that tracheostomies can be performed without prophylactic plasma transfusion and bleeding complications in critically ill patients despite increased INR in case of normal thromboelastometry (ROTEM) results. Thromboelastometry-based restrictive transfusion management helped avoid unnecessary plasma and platelet transfusion, and should reduce the incidence of transfusion-related adverse events and transfusion-associated hospital costs. Therefore, the authors believe that thromboelastometry-based strategies should be implemented to optimize patient blood management in perioperative medicine.

## Background

Thirty to Ninety percent of plasma transfused perioperative or at the ICU has to be considered as inappropriate [1–6]. Here, plasma transfusion has been considered as inappropriate in patients with normal INR (<1.5), transfused as a prophylaxis in non-bleeding patients with mildly abnormal coagulation tests, and transfused in an ineffective dose (<10 mL/kg bw). Post-transfusion corrections of INR are consistently small unless the pre-transfusion INR is only mildly deranged (<2.5) [7]. Notably, the most frequent transfused dose of FFP is two to four units, which has been shown to be ineffective to improve coagulation in several studies [6–10]. A systematic review of the literature including 80 RCTs showed no consistent evidence of significant benefit for prophylactic and therapeutic use of FFP across a range of indications evaluated [11]. Accordingly, Müller et al. [12] could not show any differences in bleeding complications in critically ill patients undergoing an invasive procedure in their RCT, regardless whether FFP was prophylactically administered or not. In trauma, even with a :PRBC transfusion ratio of 1:1 it took in mean 14.8 h to correct an INR of 1.8 ad admission to a INR of < 1.5 at the ICU [13].

On the other hand, plasma transfusion is associated with a high risk of transfusion-associated adverse events such as transfusion-associated circulatory overload (TACO), transfusion-related lung injury (TRALI), transfusion-related immunomodulation (TRIM), and anaphylaxis/allergic reactions. TRIM results in a significant increase in the incidence of nosocomial infections and sepsis in critically ill surgical patients transfused with FFP (RR, 2.99; 95 % CI, 2.28–3.93) [14]. Still, TRALI is the leading cause of transfusion-related death in the US with an incidence of TRALI/possible TRALI of 1.4 to 3.0 % of adult patients undergoing surgery and receiving transfusion [15]. Furthermore, TACO is a leading cause of transfusion-related fatalities in the US with an incidence of 3–5.5 % in transfused patients [16]. This is in line with the SHOT report 2013, reporting TACO as the leading cause of transfusion-related death (12 of 22 or 54.5 %) and major morbidity (43 of 143 or 30.1 %) in the UK [17]. Accordingly, plasma is the allogeneic blood product with the highest impact on increased long-term mortality (HR, 1.060 per unit transfused; *P* < 0.001) in patients undergoing coronary artery bypass surgery [18]. Furthermore, in non-massively transfused trauma patients (<10 U PRBC within 12 h of admission), plasma administration was associated with a substantial increase in complications, in particular ARDS (12-fold), with no improvement in survival. An increase in multiple organ dysfunction (6-fold), pneumonia (4-fold), and sepsis (4-fold) was likewise seen as increasing volumes of plasma (>6 U) were transfused [19]. In another US trauma study, exposure to ABO-compatible (pre-thawed “universal donor” plasma) versus ABO-identical plasma resulted in an increase in overall complications (53.5 % vs 40.5 %, *P* = 0.002), in particular ARDS (19.4 % vs 9.2 %, *P* = 0.0011) and sepsis (38.0 % vs 28.9 %, *P* = 0.02). There was a stepwise increase in the complication rate as exposure increases (reaching 70.0 % for patients receiving more than 6 U; these patients also had a 4-fold increase in ARDS) [20]. This is in line with a study in 86,082 Swedish patents receiving their first plasma transfusion and had a follow up of 14 days. Here, transfusion of five or more units of AB0-compatible but non-identical plasma was associated with an increased mortality (RR, 1.15; 95 % CI, 1.02–1.29) compared to recipients of only AB0-identical plasma [21]. Therefore, the avoidance of inappropriate plasma transfusion has a high potential of improving patient outcomes.

### Main text

Durila et al. [22] assessed in their prospective study, published recently in BMC Anesthesiology, whether tracheostomy in 119 septic and non-septic ICU patients can be performed without bleeding complications in case of normal thromboelastometry (ROTEM) results (EXTEM CT (coagulation time)) despite increased INR. Normal INR (≤1.2) was found in 64 (54 %) patients, while increased INR (>1.2) was found in 55 (46 %) patients. Patients with INR ≥ 1.3 (median INR, 1.48; range, 1.3–1.84) were further assessed by thromboelastometry. Despite prolonged INR, thromboelastometry results were in normal ranges in all cases except one. With normal thromboelastometry, tracheostomy was performed safely without FFP transfusion and any bleeding complication. Here, thromboelastometry analysis avoided unnecessary FFP transfusion in 98 % (54/55) critically ill patients with increased INR. Since their former practice was to transfuse four units of FFP prophylactically prior to tracheostomy in patients with an INR > 1.2, implementation of thromboelastometry testing reduced FFP transfusion from 220 to 4 units in this setting without any increase in bleeding complications. Their study provides a valuable contribution to the increasing evidence that invasive interventions, such as tracheostomy, central venous line insertion or liver biopsy, can be performed without prophylactic FFP or platelet transfusion in critically ill patients with abnormal standard laboratory tests, such as INR or platelet count, in case of normal thromboelastometry results. This viscoelastic method can help physicians to avoid unnecessary transfusion and corresponding transfusion-related adverse events.

## Discussion

Standard laboratory coagulation tests (SLTs), such as PT/INR and platelet count, are still frequently used to assess coagulopathy and to guide hemostatic interventions. However, Haas et al. recently reviewed the evidence for the usefulness of SLTs to assess coagulopathy and to guide bleeding management in the perioperative and massive bleeding setting. Evidence for the usefulness of SLTs was found in only three prospective trials, investigating a total of 108 patients, whereby microvascular bleeding was a rare finding. No data from RCTs supported the use of SLTs. In contrast, numerous investigations have challenged the reliability of SLTs to assess coagulopathy or guide bleeding management [23]. In another study, Haas et al. [24] demonstrated that PT/INR and aPTT tests overestimate the underlying coagulation factor deficiency if more than one factor is reduced, which typically occurs in dilutional coagulopathy. Thus, impaired PT/INR and aPTT values are a very common and early finding during hemodilution and intraoperative bleeding, and therefore, decision-making based on PT/INR and aPTT may lead to considerable over-transfusion of FFP and other blood products. Nevertheless, increased INR values correlate quite well with EXTEM CT in warfarin-treated patients and healthy controls (Spearman rho = 0.87). Here, EXTEM CT has a sensitivity and specificity of 0.89 and 1.00, respectively, to detect elevated INR above 1.2, with a positive and negative predictive value of 1.00 and 0.88, respectively [25]. However, the correlation between INR and EXTEM CT seem to be different in other setting, such as dilutional coagulopathy, cirrhosis, sepsis, and patients with extracorporeal assist devices [24, 26–28]. Here, tissue factor-expression on circulating monocytes can result in a shortening of CT in whole blood viscoelastic tests whereas plasmatic SLTs are unable to detect this effect [29]. In contrast to EXTEM CT, the false-negative rate of kaolin-TEG and rapid-TEG (kaolin plus tissue factor activation) for detecting warfarin coagulopathy with either test is clinically unacceptable [30]. This may be due to the fact that the intrinsic pathway is activated in these tests.

Similar considerations have been made regarding the predictive value of platelet count versus thromboelastometric clot firmness for bleeding after invasive interventions. Weigand et al. [31] demonstrated that coagulation disorders with altered INR (≥1.5) or platelet count (≤50×10^9^/L) did not increase the risk of significant bleeding when inserting a central venous catheter. Zeidler et al. [32] demonstrated in their retrospective single-center analysis of 604 central venous catheter insertions in 193 patients with acute leukemia that only patients with platelet counts of less than 20×10^9^/L were at higher risk of bleeding compared to patients with platelet counts of 100×10^9^/L or more. However, Greene et al. [33] demonstrated that thromboelastometric clot firmness (EXTEM or INTEM A10 or MCF) is superior to platelet count in predicting bleeding in pediatric patients with immune thrombocytopenia (ITP) and severe thrombocytopenia (<30×10^9^/L). Superiority of thromboelastometry maximum clot firmness (MCF) compared to platelet count in predicting bleeding in patients with hematological malignancies has recently been confirmed in the prospective observational ATHENA study [34]. Further studies confirmed that EXTEM clot firmness (A10 and MCF) can be used to assess the efficacy of prophylactic platelet transfusion before insertion of a central venous catheter in patients with bone morrow failure and in thrombocytopenic children undergoing chemotherapy [35, 36]. Furthermore, Fayed et al. [37] reported that a thromboelastometry-guided transfusion protocol in patients undergoing liver transplantation can avoid 75 % of platelet transfusions in patients with a platelet count < 50×10^9^/L without increased bleeding rate. Therefore, thromboelastometry clot firmness (EXTEM or INTEM A10 or MCF) should be considered in addition to EXTEM CT in particular in critically ill patients with thrombocytopenia undergoing tracheostomy. However, drug-, trauma- or sepsis-induced changes in platelet function cannot be detected by standard viscoelastic tests, such as ROTEM or TEG. This is an important limitation of viscoelastic testing since the high amounts of thrombin generated in these test systems can overwhelm the inhibition of other platelet activation pathways, such as the arachidonic acid, collagen, serotonin, or ADP pathway. This diagnostic gap can be closed by point-of-care platelet function testing such as whole blood impedance aggregometry (Multiplate or ROTEM platelet) which is much more sensitive to these effects [28, 38–40]. Platelet dysfunction cannot only be induced by so call “antiplatelet drugs” but also by a lot of other drugs used for treatment in critically ill patients such as non-steroidal anti-inflammatory drugs (NSAIDs), selective serotonin release inhibitors (SSRIs), β-lactam antibiotics, as well as cardiovascular and lipid-lowering drugs [41, 42]. In particular combination of these drugs can induce severe platelet dysfunction [42, 43].

Accordingly, point-of-care-guided bleeding management algorithms, using thromboelastometry and impedance aggregometry, have been shown to be effective in reducing transfusion requirements in cardiovascular surgery, liver transplantation, trauma, obstetrics, and other critically ill patients [44–48]. Here, transfusion requirements for FFP, PRBC, and platelets could be reduced by 70–90 %, 10–60 %, and 20–70 %, respectively [49]. In addition, this is associated with significant cost-savings [48–51]. Furthermore, the incidence of massive transfusion, revision surgery, TACO, acute lung injury, multiple organ failure, nosocomial infection and sepsis, as well as thromboembolic events could be reduced significantly [44, 47–53]. The later can be explained by the high predictive value of thromboelastometry for postoperative complications after major non-cardiac surgery (ROC AUC = 0.751 for INTEM A10), which allows for defining a therapeutic window to stop bleeding without increased risk of thromboembolic events [54]. Similar results have been reported for cardiovascular patients [55].

## Conclusions

The prospective study by Durila et al. [22] provides further evidence that invasive interventions such as tracheostomy can be performed without prophylactic plasma transfusion and bleeding complications in critically ill patients with increased INR in case of normal thromboelastometry results. thromboelastometry-based restrictive transfusion management, avoiding unnecessary plasma and platelet transfusion, can reduce the incidence of transfusion-related adverse events and transfusion-associated hospital costs. Therefore, the authors believe that thromboelastometry-based strategies should be implemented to optimize patient blood management in perioperative medicine.

## Abbreviations

A10: Amplitude of clot firmness 10 min after CT
aPTT: Activated partial thromboplastin time
ARDS: Acute respiratory distress syndrome
bw: Body weight
CI: Confidence interval
CT: Coagulation time
EXTEM: Extrinsically (tissue factor) activated ROTEM assay
FFP: Fresh frozen plasma
HR: Hazard ratio
ICU: Intensive care unit
INR: International normalized ratio
INTEM: Intrinsically (ellagic acid) activated ROTEM assay
ITP: Idiopathic thrombocytopenic purpura
MCF: Maximum clot firmness
PRBC: Packed red blood cells
PT: Prothrombin time
RCT: Randomized controlled trial
ROTEM: Rotational thromboelastometry
RR: Relative risk
SHOT: Serious hazards of transfusion
TACO: Transfusion-associated circulatory overload
TEG: Thrombelastography
TRALI: Transfusion-related acute lung injury
TRIM,1: Transfusion-related immunomodulation
